# Measuring Health Insurance Literacy in the Netherlands – first results of the HILM-NL questionnaire

**DOI:** 10.1093/eurpub/ckac129.346

**Published:** 2022-10-25

**Authors:** L Holst, JJDJM Rademakers, AEM Brabers, JD de Jong

**Affiliations:** 1 Healthcare System and Governance, NIVEL, Utrecht, Netherlands; 2 CAPHRI, Maastricht University, Maastricht, Netherlands

## Abstract

**Background:**

Many Western countries have healthcare systems that expect their citizens to make critical, well-considered decisions about which health insurance policy best fits their needs and preferences. There are indications that citizens struggle to fulfil this role due to the complexity of the healthcare systems and lack of skills, support or motivation. This can lead to citizens being sub-optimally insured, suffering inadequate coverage and facing unexpected costs. To understand how citizens choose and use a policy, the health insurance literacy measure (HILM) was developed in the US. It seems valuable to investigate the concept of Health Insurance Literacy (HIL) in other countries. This study aims to examine how HIL is distributed among citizens in the Netherlands; and to find out whether certain groups have more difficulty choosing and using a health insurance policy.

**Methods:**

We measured HIL using the HILM-NL questionnaire, a validated and translated version of the HILM. In February 2020, the HILM-NL was sent to 1,500 members of the Nivel Dutch Health Care Consumer Panel. The response rate was 54% (n = 806). Higher HILM-NL scores imply a higher self-assessed ability in choosing and using health insurance.

**Results:**

There is a wide variation in HIL among citizens in the Netherlands. The average total HILM-NL score is 55.14 (ranging from 21-84). Lower-educated citizens (p<.04) and citizens with lower income (p<.01) are relatively more likely to have lower HIL, than, respectively, higher-educated citizens and citizens with higher income.

**Conclusions:**

Citizens who completed less education or earn a lower income are relatively more likely to have difficulty choosing and using a health insurance policy. It is important to support these vulnerable groups, so that Dutch citizens in general will be better able to choose a policy that fits their needs and preferences. This should ensure that citizens are less likely have to deal with inadequate coverage and unexpected costs.

**Key messages:**

• There is a wide variation in HIL among citizens in the Netherlands.

• Citizens who completed less education or earn a lower income are relatively more likely to have difficulty choosing and using a health insurance policy.

